# The Japanese as Sanitary Reformers

**Published:** 1899-01

**Authors:** 


### The Japanese as Sanitary Reformers.

The Japanese government is said to have ordered the complete abandonment of the city of Leck Cham, on the island of Formosa, which has frequently been subject to epidemics of such malignancy that thousands of lives were lost. While Formosa belonged to China, no effort was made to ascertain or remove the cause. The Japanese have been in possession of the island for about three years, and soon after it passed into their hands they set themselves to discover the cause of the unhealthiness of Leck Cham. A committee of expert sanitarians reported that the city was built on a swamp from which poisonous gases came to the surface, and that the city was so thoroughly permeated with disease germs that disinfection was impossible. The government thereupon issued an order that a new and healthy site should be selected as near the old site as was prudent. Such a site was recommended and accepted, and was laid out by experts, with sewers, streets, pavements, waterworks, and all modern improvements, without any expense to the inhabitants of the old city. Every property holder in the old city, without expense to himself, was assigned the same area of ground in the new Leck Cham, and he was given twelve months in which to remove himself and belongings to his new abode. The result of this drastic experiment will be awaited with interest, though it can hardly be doubted that it will be highly successful if the thoroughness of the local authorities, in the matter of scavenging in the future corresponds at all with that of the central authority.—*British Medical Journal.*

Even the Japs are ahead of Texas.

